# Elevated Exposure to Air Pollutants Accelerates Primary Glomerular Disease Progression

**DOI:** 10.1016/j.ekir.2024.05.013

**Published:** 2024-05-18

**Authors:** Jonathan P. Troost, Jennifer D’Souza, Miatta Buxton, Abhijit V. Kshirsagar, Lawrence S. Engel, Cassandra R. O’Lenick, William E. Smoyer, Jon Klein, Wenjun Ju, Sean Eddy, Margaret Helmuth, Laura H. Mariani, Matthias Kretzler, Howard Trachtman

**Affiliations:** 1 Michigan Institute for Clinical & Health Research, University of Michigan, Ann Arbor Michigan, USA; 2 Department of Epidemiology, University of Michigan, Ann Arbor, Michigan, USA; 3 Division of Nephrology and Hypertension, Department of Medicine, University of North Carolina, Chapel Hill, North Carolina, USA; 4 Departments of Epidemiology and Environmental Sciences and Engineering, University of North Carolina, Chapel Hill, North Carolina, USA; 5 Department of Pediatrics, Ohio State University, The Abigail Wexner Research Institute at Nationwide Children’s Hospital, Columbus, Ohio, USA; 6 Division of Nephrology and Hypertension, Department of Medicine, Christina Lee Brown Environment Institute, University of Louisville School of Medicine, Louisville, Kentucky, USA; 7 Robley Rex Veterans Administration Medical Center, Louisville, Kentucky, USA; 8 Department of Internal Medicine, University of Michigan, Ann Arbor, Michigan, USA; 9 Department of Computational Medicine and Bioinformatics, University of Michigan, Ann Arbor Michigan, USA; 10 Division of Nephrology, Department of Pediatrics, University of Michigan, Ann Arbor, Michigan, USA

**Keywords:** black carbon, exposures, kidneys, PM2.5, progression, sulfates

## Abstract

**Introduction:**

Environmental contributors to kidney disease progression remain elusive. We explored how residential air pollution affects disease progression in patients with primary glomerulopathies.

**Methods:**

Nephrotic Syndrome Study Network (NEPTUNE) and CureGlomerulonephropathy (CureGN) participants with residential census tract data and ≥2 years of follow-up were included. Using Cox proportional hazards models, the associations per doubling in annual average baseline concentrations of total particulate matter with diameter ≤2.5 μm (PM_2.5_) and its components, black carbon (BC), and sulfate, with time to ≥40% decline in estimated glomerular filtration rate (eGFR) or kidney failure were estimated. Serum tumour necrosis factor levels and kidney tissue transcriptomic inflammatory pathway activation scores were used as molecular markers of disease progression.

**Results:**

PM_2.5_, BC, and sulfate exposures were comparable in NEPTUNE (*n* = 228) and CureGN (*n* = 697). In both cohorts, participants from areas with higher levels of pollutants had lower eGFR, were older and more likely self-reported racial and ethnic minorities. In a fully adjusted model combining both cohorts, kidney disease progression was associated with PM_2.5_ (adjusted hazard ratio 1.55 [95% confidence interval: 1.00–2.38], *P* = 0.0489) and BC (adjusted hazard ratio 1.43 [95% confidence interval: 0.98–2.07], *P* = 0.0608) exposure. Sulfate and PM_2.5_ exposure were positively correlated with serum tumour necrosis factor (TNF) (*P* = 0.003) and interleukin-1β levels (*P* = 0.03), respectively. Sulfate exposure was also directly associated with transcriptional activation of the TNF and JAK-STAT signaling pathways in kidneys (r = 0.55–0.67, *P*-value <0.01).

**Conclusion:**

Elevated exposure to select air pollutants is associated with increased risk of disease progression and systemic inflammation in patients with primary.

## Graphical abstract

**Figure:**
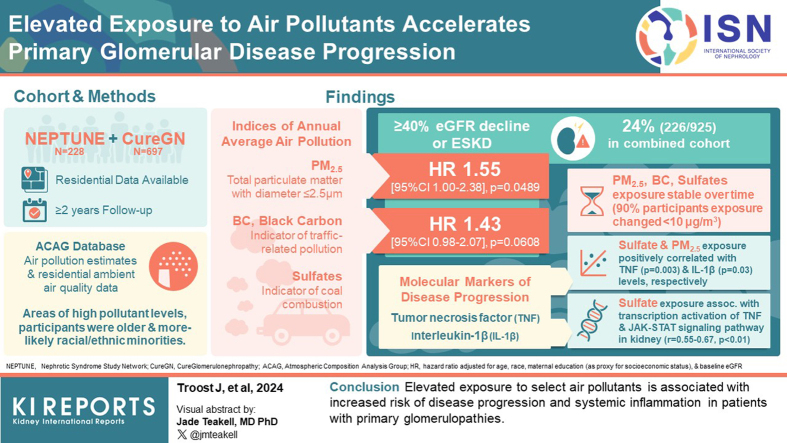

Air pollution is a global health problem linked to chronic conditions, including hypertension,^1^ cardiovascular and pulmonary disease.^2^, ^3^, ^4^, ^5^ It stimulates multiple injury pathways, including oxidant stress and autonomic imbalances and causes vascular dysfunction, all of which can contribute to target organ damage.^6^ Common air pollutants include ozone, nitrogen oxide, sulfur dioxide, and fine particulate matter <2.5 μm in diameter (PM_2.5_). PM_2.5_ is generated by the combustion of solid and liquid fuels, and its chemical composition varies geographically based on combustion sources and meteorology.^7^^,^^8^ The major PM_2.5_ constituents include metal oxides, sulfates, organic carbon, and elemental or BC.^9^ There is evidence suggestive of differential toxicity of PM_2.5_ depending on the source and chemical composition.^6^^,^^10^

Evidence for adverse effects on cardiovascular and kidney function from exposure to PM_2.5_ include the association with increased blood pressure, and albuminuria and decreased GFR.^11^ Limited data demonstrate that exposure to PM_2.5_ is associated with an increased risk of incident chronic kidney disease and its progression to kidney failure.^12^^,^^13^ Black, Hispanic, and socioeconomically marginalized communities, which are disproportionately affected by chronic kidney disease, are also often exposed to higher concentrations of PM_2.5_ because of a history of racially discriminatory practices and policies, such as redlining and placement of transportation infrastructure that affected place of residence and proximity to industrial sources of air pollution, respectively.^11^, ^12^, ^13^, ^14^, ^15^, ^16^

Emerging evidence suggests a role for PM_2.5_ in the pathogenesis of glomerular diseases. In a study based in China, PM_2.5_ levels in outdoor air were associated with an increased risk of membranous nephropathy^17^ but not with other common glomerulopathies, whereas another report documented an increased risk of disease progression in adults with IgA nephropathy.^18^ Both of these studies focused on total PM_2.5_ but did not examine the potential impact of its components–an important gap because particles are generated from a range of sources, including windblown dust, sea salt, as well as traffic and coal-fired power plants, and may have differential toxicities.^6^ Additionally, we are not aware of any studies on air pollution and the rate of disease progression in patients with primary glomerulopathies, inclusive of membranous nephropathy, IgA nephropathy, minimal change disease (MCD) and focal segmental glomerulosclerosis. Finally, no studies have explored the potential inflammatory pathways involved in air-pollution-induced kidney injury.

Therefore, this study was conducted in well-characterized patients with membranous nephropathy, IgA nephropathy, MCD, and focal segmental glomerulosclerosis enrolled in the NEPTUNE and CureGN observational cohorts to test the following 2 hypotheses: (i) higher levels of residential PM_2.5_, based on census tract level data are associated with increased risk of disease progression and (ii) the level of exposure is associated with the serum concentration of proinflammatory biomarkers and with intrarenal inflammatory pathway signaling. To understand the potential impacts of various sources of PM_2.5_, we evaluated relationships with total PM_2.5_, BC (indicator of diesel traffic), and sulfate (indicator of coal combustion).

## Methods

### Patients

All patients enrolled in the prospective cohort studies, NEPTUNE and CureGN, from 2010 to 2022 in any of the 5 defined disease subgroups membranous nephropathy, IgA nephropathy, MCD, focal segmental glomerulosclerosis, and paediatric patients with nephrotic syndrome without biopsy–with available valid and confirmed census tract-level address data and who have been followed for ≥2 years were included in this study.^19^^,^^20^ Enrollment into NEPTUNE was at the time of a diagnostic biopsy performed for the evaluation of proteinuria or within 30 days of new-onset nephrotic syndrome in pediatric participants without biopsy. This reflects the standard of care for children who are presumed to have MCD and are treated empirically with corticosteroids. CureGN includes prevalent patients with a biopsy-confirmed diagnosis of primary glomerular disease within 5 years of enrollment. Both cohorts include pediatric and adult patients. This minimum duration of observation was set to enable assessment of chronic exposure to the air pollutants and to improve the accuracy of the calculation of the decline in GFR.^21^, ^22^, ^23^

The following study baseline data were tabulated from the NEPTUNE and CureGN database: eGFR, urine protein:creatinine ratio, age, sex, race/ethnicity, maternal education (less than high school, completed high school, or college or more) as a proxy for socioeconomic status,^24^ diagnosis, and census tract location of residence at study enrolment. This information was collected at baseline, at each scheduled follow-up visit per study protocols, i.e., every 6 months in NEPTUNE and every 4 to 6 months in CureGN, and retrospectively to the date of biopsy for CureGN. Race is self-reported or reported by parents of children and reporting was mandated by National Institutes of Health.

### Air Pollution Data

The following 3 indices of annual average residential air pollution exposure were assessed: (i) PM_2.5_, which reflects total fine particle levels from all sources; (ii) BC, which is commonly used as an indicator for traffic-related pollution; and (iii) sulfates, which are used as an indicator of coal combustion. Major contributors to sulfate formation include coal fired power plants, and gasoline and diesel fuels containing sulfur.

To estimate ambient air quality for our study participants by residential census tract, we leveraged a publicly available database (https://sites.wustl.edu/acag/datasets/surface-pm2-5/) of estimated PM_2.5_ concentrations available in annual and monthly average estimates at high spatial resolution 0.01° × 0.01° (approximately 1 km × 1 km). These data have been derived by combining Aerosol Optical Depth retrievals from NASA satellites (MODIS, MISR, and SeaWIFS) with the GEOS-Chem chemical transport model, and subsequently calibrated to ground-based observations using a geographically weighted regression. All NEPTUNE and CureGN sites from the United States were included in this study.

Census-tract level averages for the exposures were calculated for each participant for the calendar year of every visit. However, given the small degree of within-participant changes over time (Supplementary Figure S1A–C), only air pollution estimates from the year before enrollment (baseline exposure) were used in analyses. Annual air pollution estimates for PM_2.5_ were available from 2000 to 2018; BC and sulfates estimates were available from 2000 to 2016.

### Laboratory Methods

Plasma TNF and interleukin-1β (IL-1β) concentrations were measured using Slow Off-rate Modified Aptamer–based protein identification by SomaLogic (Boulder, CO). The quantification, assay calibration and normalization were performed following the standard laboratory protocol. Log2 transformed expression values were derived from quantile-normalized data files and used for correlation analysis. A subset of NEPTUNE participants had census tract data and cytokine measurements available (*n* = 15–19).

### Transcriptome Profiling

In NEPTUNE, RNA sequencing was performed on manually micro dissected kidney biopsy tissue that separated tubulointerstitial and glomerular compartments of the research core. Gene expression activation of JAK-STAT and TNF activated pathways was calculated using their downstream transcriptional activation profiles for both the glomerular and tubular compartments, as previously described.^25^^,^^26^ A subset of NEPTUNE participants had census tract and tissue transcriptomic data (*n* = 29).

### Statistical Methods

Continuous data were described using medians, interquartile ranges, and the first and third quantiles. Categorical variables were described using frequencies and percentages. The frequencies of missing variables are provided in Table 1, Table 2. We tested for multiplicative interaction between air pollution exposures and patient age, primary glomerular disease, and time from kidney biopsy to study enrollment (<1 year vs. ≥1 year) and kidney outcomes. We used Kruskal-Wallis and χ^2^ tests when comparing characteristics by disease duration and cohort. When comparing by levels of environmental exposure, we treated the environmental exposure as a continuous variable and thus used Pearson correlation for continuous∗continuous comparisons *P*-values and Kruskal-Wallis for continuous∗categorical comparisons. Complete case analysis was performed, and no imputation was done for missing data. The frequencies of missing variables are shown in Table 1, Table 2 and Supplementary Tables S3 and S4. The median split was done to help tabulate the data.

**Table 1:** Descriptive characteristics in the overall combined NEPTUNE and CureGN cohort and by comparison of PM_2.5_ exposure (μg/mm^3^) at baseline above and below the median level of exposure

Characteristic	aOverall (*n* = 925)	PM2.5 ≤ median (≤8.26) (*n* = 461)	PM2.5 > median (>8.26) (*n* = 464)	*P*-value
PM_2.5_ at baseline, median (IQR)	8.0 (7.0–9.1)	7.0 (6.3–7.4)	9.1 (8.5–9.9)	
Black carbon at baseline, median (IQR)	0.7 (0.6–0.9)	0.6 (0.5–0.7)	0.8 (0.7–1.0)	<0.001
Sulfate (SO_4_) at baseline, median (IQR)	1.5 (1.2–1.7)	1.3 (1.0–1.6)	1.6 (1.3–1.9)	<0.001
Age at baseline (yr), median (IQR)	21.0 (9.0–48.0)	17.0 (8.0–48.0)	26.0 (10.0–47.5)	0.02
Sex, *n* (%)				0.85
Male	516 (56)	260 (56)	256 (55)	
Female	409 (44)	201 (44)	208 (45)	
Race, *n* (%)				<0.001
Asian/Asian American	57 (6)	24 (5)	33 (7)	
Black/African American	204 (22)	79 (17)	125 (27)	
Multi-Racial	41 (4)	16 (3)	25 (5)	
Native Hawaiian/Other Pacific Islander	6 (1)	4 (1)	2 (0)	
White/Caucasian	584 (63)	322 (70)	262 (56)	
Unknown	33 (4)	16 (3)	17 (4)	
Ethnicity, *n* (%)				<0.001
Hispanic or Latino	157 (17)	53 (11)	104 (22)	
Not Hispanic or Latino	762 (82)	406 (88)	356 (77)	
Unknown	6 (1)	2 (0)	4 (1)	
Maternal education, *n* (%)				<0.001
Unknown	92 (10)	36 (8)	56 (12)	
<High school	204 (22)	91 (20)	113 (24)	
High school	401 (43)	215 (47)	186 (40)	
≥4-yr college	228 (25)	119 (26)	109 (23)	
Diagnosis, *n* (%)				0.04
FSGS	203 (22)	86 (19)	117 (25)	
IgA nephropathy	202 (22)	104 (23)	98 (21)	
MCD	235 (25)	128 (28)	107 (23)	
MN	163 (18)	87 (19)	76 (16)	
Non-biopsied	106 (11)	46 (10)	60 (13)	
Other	16 (2)	10 (2)	6 (1)	
eGFR at baseline, median (IQR)	87.9 (57.3–111.7)	89.8 (61.3–111.5)	84.2 (52.9–111.8)	0.002
UPCR at baseline, median (IQR)	1.8 (0.2–6.6)	1.6 (0.2–6.6)	2.0 (0.3–6.5)	0.98
Follow-up time (mo), median (IQR)	49.0 (27.1–67.1)	50.1 (31.0–66.9)	46.0 (19.3–68.2)	0.03

eGFR, estimated glomerular filtration rate; FSGS, focal segmental glomerulosclerosis; IQR, interquartile ranges; MCD, minimal change disease; MN, membranous nephropathy; PM_2.5_, particulate matter with diameter ≤ 2.5μm; UPCR, urine protein:creatinine ratio.

a *N* missing per continuous variables: age = 0, eGFR = 53; UPCR = 107; PM_2.5_ = 0; Black carbon = 350; Sulfate = 350; Follow-up time = 0.

**Table 2:** Unadjusted and adjusted risk of ≥40% decline in eGFR or ESKD associated with exposure to log transformed levels of exposure to the three air pollution components in the pooled NEPTUNE and CureGN cohort

POOLED (NEPTUNE+CureGN)	PM_2.5_^a^		Black carbon		Sulfate (So4)	
	Estimate (95% CI)	*P*-value	Estimate (95% CI)	*P*-value	Estimate (95% CI)	*P*-value
Unadjusted						
Environmental marker at baseline (per doubling)	1.69 (1.15–2.49)	0.0079	1.60 (1.18**–**2.16)	0.0025	1.17 (0.86**–**1.59)	0.3132
Adjusted-1						
Environmental marker at baseline (per doubling)	1.59 (1.03**–**2.45)	0.0347	1.47 (1.01**–**2.14)	0.0446	1.06 (0.76**–**1.47)	0.7479
Age (per 1 yr)	1.00 (0.99**–**1.01)	0.7560	1.01 (1.00**–**1.02)	0.0668	1.01 (1.00**–**1.02)	0.0447
Race: Black vs. non-black	1.59 (1.16**–**2.19)	0.0045	2.14 (1.42**–**3.21)	0.0003	2.28 (1.51**–**3.43)	<.0001
Maternal education	1.51 (1.06**–**2.13)	0.0211	1.93 (1.23**–**3.01)	0.0040	1.74 (1.13**–**2.69)	0.0120
High school vs. <High school	0.82 (0.52**–**1.27)	0.3713	1.11 (0.63**–**1.96)	0.7255	1.01 (0.58**–**1.78)	0.9623
College vs. <High school	0.83 (0.53**–**1.29)	0.3985	1.15 (0.64**–**2.04)	0.6446	1.01 (0.58**–**1.78)	0.9647
Adjusted-2						
Environmental marker at baseline (per doubling)	1.55 (1.00**–**2.38)	0.0489	1.43 (0.98**–**2.07)	0.0608	0.98 (0.69**–**1.37)	0.8883
Age (per 1 yr)	0.99 (0.98**–**1.00)	0.0015	0.99 (0.98**–**1.00)	0.1586	0.99 (0.98**–**1.00)	0.1906
eGFR at baseline (per 1 ml)	0.98 (0.98**–**0.99)	<.0001	0.98 (0.97**–**0.99)	<.0001	0.98 (0.97**–**0.99)	<.0001
Race: Black vs. non-black	1.27 (0.91**–**1.76)	0.1600	1.66 (1.08**–**2.53)	0.0195	1.73 (1.13**–**2.65)	0.0117
Maternal education						
High school vs. <High school	1.46 (1.03**–**2.06)	0.0339	1.80 (1.15**–**2.82)	0.0099	1.64 (1.06**–**2.52)	0.0257
College vs. <High school	0.83 (0.53**–**1.29)	0.4126	1.10 (0.62**–**1.94)	0.7452	1.01 (0.58**–**1.78)	0.9597

CI, confidence interval; eGFR, estimated glomerular filtration rate.

a Data from 53 participants were excluded for missing eGFR in the PM_2.5_ model and 30 were excluded in the black carbon and sulfate models.

Cox proportional hazards models were used to analyze time from baseline to either end stage kidney disease (ESKD) diagnosis or *≥*40% reduction in eGFR from baseline, a shared endpoint used in both the NEPTUNE and CureGN cohorts. We confirmed that Schoenfeld residuals were not correlated with time. For each marker, we performed an unadjusted model, a model adjusted for age, race, and maternal education as a proxy for socioeconomic status, and a second model adjusted for the above factors and eGFR at baseline. We report our findings per standard deviation difference to aid in interpretability across pollutants. Kaplan-Meier plots were created to illustrate the time to the kidney outcome based on the quartile level of baseline exposure to the 3 air pollutants.

An assessment of the relationship among the 3 components of air pollution and baseline serum concentration of TNF and tissue transcriptomic pathway activation scores in NEPTUNE was made using Pearson correlation coefficients and scatter plots. Analyses were performed using SAS v9.4 (SAS Institute Inc., Cary, NC).

## Results

The NEPTUNE and CureGN cohorts differed in age and timing of study entry relative to disease onset. CureGN represented a more heterogeneous group in terms of time since biopsy, some of whom were enrolled shortly after a diagnostic biopsy and others who were enrolled later in the 5-year post biopsy eligibility window (Supplementary Table S1). When we tested for interaction between exposure to the 3 components of air pollution and patient age, underlying disease, and interval between diagnostic kidney biopsy and study enrollment on the kidney outcome, none of the terms were significant. In addition, we observed no clinically meaningful differences between the populations enrolled in the 2 observational studies, e.g., by patient demographics or overall disease severity. Therefore, the 2 cohorts were combined to create a larger population of patients with glomerular disease and to potentially increase the power to detect associations between air pollution exposure and the progression of kidney disease. The characteristics of the combined patient population are summarized in Table 1.

The baseline levels of exposure to the 3 individual air pollutants were significantly correlated with 1 another (Supplementary Table S2), but correlation was weakest between sulfates and the other 2 pollutants. For all 3 classes of air pollutants, patients with exposures above the median were older, more likely to be Black, and had a lower eGFR at baseline (*P* < 0.005). For each individual pollutant, those with exposure above the median had higher levels of exposure to the other 2 pollutants, than participants with exposure below the median (Table 1, Supplementary Tables S3 and S4).

The exposures to PM_2.5_, BC, and sulfates were relatively stable over time, and 90% of participants had exposures that changed by <10 μg/m^3^ (Supplementary Figure S1–C). Moreover, the same percentage had a <25% difference between their highest and lowest values of exposure to the 3 air pollution components. Therefore, we assessed the association between baseline levels of exposure (year before enrollment) to the specific pollutants and kidney function outcomes. Data were log transformed to account for the skewed distribution. Overall, 226 of 925 participants (24%) in the combined NEPTUNE-CureGN cohort developed a 40% decline in eGFR or progressed to ESKD. Evaluating the full cohort, in unadjusted analyses, a doubling of baseline PM_2.5_ and separately, BC, was associated with a greater risk of disease progression, with HR of 1.69 (1.15–2.49) (*P* = 0.0079) and 1.60 (1.18–2.16) (*P* = 0.0025), respectively. The associations with PM_2.5_ and BC persisted, PM_2.5_ HR 1.55 (1.00–2.38) (*P* = 0.0489) and BC HR 1.43 (0.98–2.07) (*P* = 0.0608) in a fully adjusted analysis that included age, baseline eGFR, race, and maternal education (Table 2). There was no association with sulfates in unadjusted or adjusted analyses. The analysis was repeated using the untransformed data and yielded comparable results. Thus, in unadjusted analyses, 1 SD higher baseline PM_2.5_ and separately, BC, was associated with a greater risk of disease progression, HR of 1.18 (1.04–1.34) (*P* = 0.0097) and 1.24 (1.08–1.42) (*P* = 0.0026), respectively. The association with PM_2.5_ and BC persisted, PM_2.5_ HR 1.15 (0.99–1.32) (*P* = 0.0602) and BC HR 1.19 (1.00–1.42) (*P* = 0.0445) in a fully adjusted analysis (Supplementary Table S5).

**Figure 1:**
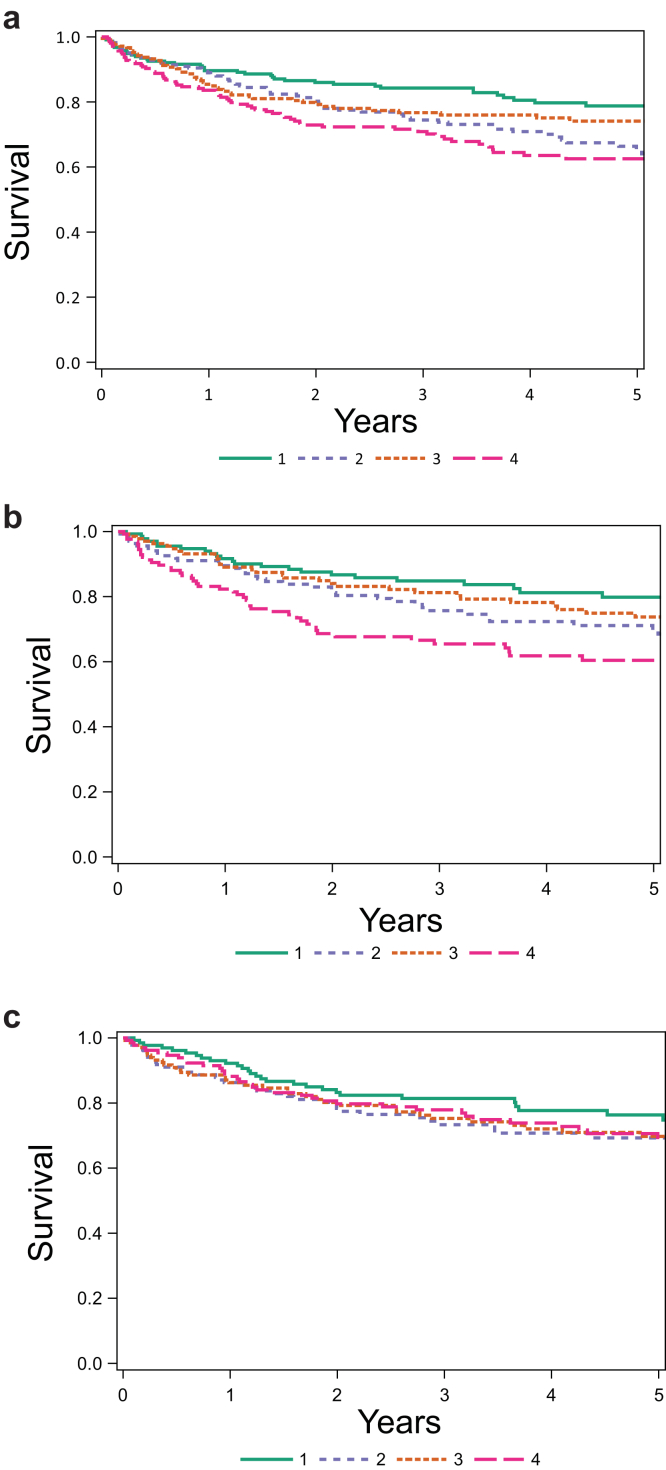
Kaplan-Meier plots illustrating the time to the kidney outcome based upon the quartile level of baseline exposure to (a) PM_2.5_; (b) Black carbon; and (c) Sulfates.

In a time to event analysis, the likelihood of manifesting a 40% reduction in eGFR or reaching ESKD was generally at higher quartile levels of exposure for all 3 air pollutants with stronger associations with PM_2.5_, and BC compared with sulfates (Figure 1a–c).

**Figure 2:**
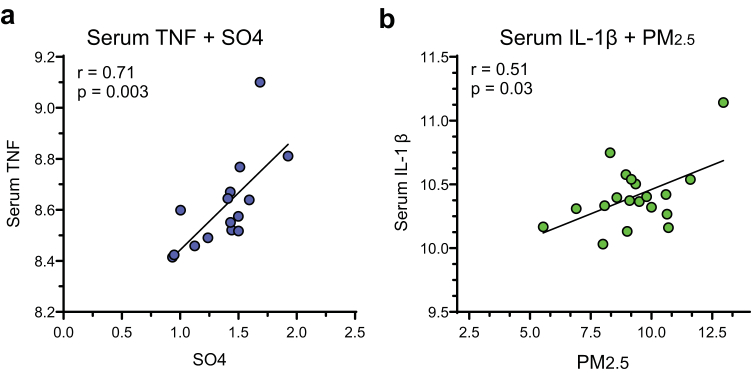
Relationship between (a) serum TNF and exposure to sulfates (*n* = 15); and (b) serum IL-1β and exposure to PM_2.5_ (*n* = 19).

**Figure 3:**
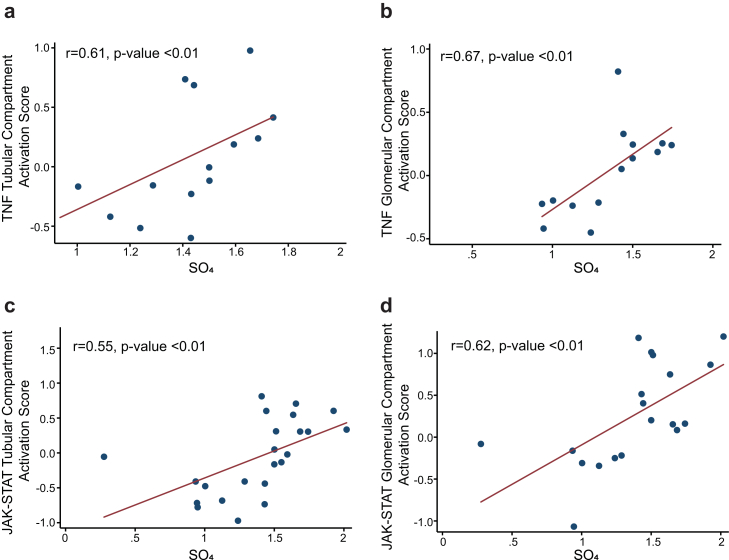
Correlation between SO4 Levels and Kidney Tissue JAK-STAT and TNF Pathway Gene Expression Activation Scores. Shown are transcript scores in both the glomerular and tubular compartments (*n* = 29). (a) TNF tubular compartment activation score and exposure to sulfates; (b) TNF glomerular compartment activation score and exposure to sulfates; (c) JAK-STAT tubular compartment activation score and exposure to sulfates; (d) JAK-STAT glomerular compartment activation score and exposure to sulfates.

The circulating level of TNF was associated with the degree of exposure to sulfates (r = 0.71, *P* = 0.003) (Figure 2a) whereas the serum concentration of IL-1β was directly related to PM_2.5_ exposure (r = 0.51, *P* = 0.03) (Figure 2b). Sulfate exposure was positively correlated with scores of gene expression activation of the TNF and JAK-STAT signaling pathways in both the glomerular and tubular tissue compartments (Figure 3). There was no correlation in this limited sample size with PM_2.5_ or BC.

## Discussion

In 2 observational cohorts of pediatric and adult patients with primary glomerular disease, NEPTUNE and CureGN, we report similar levels of exposure to 3 known air pollutants, PM_2.5_, BC, and sulfate, which were generally stable over time and were moderately correlated with one another (r = 0.37–0.71). Patients with higher levels of exposure to each of the 3 air pollutants tended to be older, were more likely to be from a racial or ethnic minority group, have a lower eGFR, and had exposures that were above the median for the other 2 pollutants. This suggests that there may be a subset of patients with glomerular disease who have a greater susceptibility to potential adverse consequences from this environmental exposure and is consistent with the reports of a social inequity in air pollution exposure. The key finding is that in the overall combined cohort of patients enrolled in NEPTUNE or CureGN, in adjusted analyses, exposure to PM_2.5_ (HR 1.55 [1.00–2.38] per doubling) and BC (HR 1.43 [0.98–2.07] per doubling) were associated with an increased risk of disease progression. The findings were comparable when the analyses were performed on untransformed exposure levels and the HR was expressed per 1-SD increase in the level. The time to event analysis were consistent and demonstrated stronger trends for earlier time to the kidney outcomes based on quartile levels of exposure to PM_2.5_, and BC compared with sulfates. We did not find a different association by disease diagnosis, age, or duration of disease. Finally, sulfate, but not BC or total PM_2.5_, exposure was positively correlated with the circulating level of TNF and intrarenal gene activation in the JAK-STAT and TNF signaling pathways in the glomerular and tubulointerstitial compartments.

Overall, the magnitude of the effects of PM_2.5_ and BC exposure assessed by HR were consistently >1. The HR for the combined end point of a ≥40% decline in eGFR or ESKD was similar in magnitude in our study of patients with primary glomerular disease compared with that in a large cohort of ∼2.5 million US veterans, with HRs in the range of 1.2 to 1.3 for eGFR < 60 ml/min per 1.73 m^2^, chronic kidney disease, eGFR decline ≥30%, and ESKD, based on baseline or time-varying exposure.^12^ Of note, we detected an association between exposure to air pollutants and adverse kidney outcomes in a sample of 925 patients, much smaller than those in most population-based studies. The susceptibility to an adverse impact of air pollution on renal function may, in fact, be greater in patients with kidney disorders because the elevated risk was noted based on a 1-SD increase in exposure to individual air pollutants rather than the larger absolute increase of 10 μg/m^3^ in PM_2.5_ concentration used in previously published reports in the general population that showed similar magnitudes of risk.^4^^,^^12^^,^^13^^,^^17^^,^^18^ This was true for the log transformed analysis as well because the increment based on doubling of exposure was generally <10 μg/m^3^. In addition, associations with adverse kidney outcomes were documented even though most of the mean levels of exposure were below the US Environmental Protection Agency’s recommended safe levels (i.e., PM_2.5_ <12 μg/m^3^). This finding aligns with the current state of the air pollution-health literature,^27^ which suggests that there is no safe threshold for PM_2.5_.

The adverse effects of air pollution on cardiovascular outcomes have been well-documented in various countries, including in patients with ESKD.^28^^,^^29^ Cardiac and kidney disease share common pathophysiological pathways of organ injury including systemic inflammation, oxidative stress, and endothelial damage. To date, few studies have analyzed the relationship between biomarkers and air pollution in patients with kidney disease. A recent study demonstrated an inverse association of PM_2.5_ and serum albumin and hemoglobin (negative acute phase reactants) among patients receiving maintenance hemodialysis.^30^ The previously described relationship between BC exposure and biomarkers of inflammation,^31^^,^^32^ and kidney disease progression observed in our study of the NEPTUNE and CureGN cohorts is consistent with a role for systemic inflammation and oxidative stress,^33^ given that increased urinary levels of MCP-1, IL1-β, and TNF have previously been linked to chronic kidney disease progression in pediatric patients.^34^ Our data associating air pollution with biomarkers of inflammation as well as intrarenal gene expression activation in the TNF and JAK-STAT signaling pathways, albeit in a limited subsample, lend support for a similar molecular mechanism of injury in kidney disease and heart disease.

The relationships between air pollution exposure and inflammatory cytokine levels and the intrarenal transcriptomic profiles indicative of JAK-STAT and TNF activation were documented in a small subset of NEPTUNE participants. As noted above, patients in NEPTUNE cohort were enrolled earlier in their disease course, tended to be younger, and had less advanced kidney disease compared to those in CureGN. This suggests that air pollution-induced inflammation may be present even in the incipient stages of glomerular disease. The clinical signal for an adverse effect on kidney function was stronger for PM_2.5_ and BC compared to sulfate exposure. In contrast, although there was a relationship between PM_2.5_ exposure and serum IL-1β levels, we also documented associations between sulfate exposure and circulating cytokine levels and intrarenal inflammatory pathway signaling. Evaluation of these relationships in expanded patient cohorts with both completed cytokine and transcriptomic assays and available air pollution exposure data is warranted.

We acknowledge the modest sample size of the 2 individual cohorts and the pooled population in this analysis. The 2 cohorts differ with NEPTUNE more reflective of incident patients enrolled at the time of their diagnostic kidney biopsy and patients in CureGN cohort having identified disease within 5 years of enrollment. However, there was no interaction between the effects of the air pollution exposures on outcomes with patient age, specific glomerular disease, or time from kidney biopsy to enrollment in 1 of the cohort studies. NEPTUNE includes pediatric patients who are eligible for inclusion in the study within 30 days of onset of nephrotic syndrome without a kidney biopsy and who are presumed to have MCD. Future work using a larger number of patients in NEPTUNE and CureGN with confirmed and validated residential census tract data will expand the dataset and allow more rigorous evaluation of the key findings. Comparison with other glomerular disease cohorts from different global regions will further clarify the impact of local air pollution exposure. This effort will enable more detailed investigation of the effects of air pollution on disease progression in the specific glomerulopathies, and the interaction with diet and other lifestyle factors, detailed residential and occupational history, socioeconomic factors, exposure to organic pollutants,^35^ genetic profiles, comorbid conditions, and treatment.

In conclusion, in an analysis of a pooled sample of 2 well-characterized cohorts of patients with primary glomerular disease, NEPTUNE and CureGN, we have documented an increased risk of disease progression in relation to levels of exposure to the PM_2.5_ and BC components of air pollution. The adverse effect of these air pollutants may be mediated by induction of increased intrarenal and systemic inflammation. Based on our findings, we recommend further study to determine (i) the effect of the specific components of air pollution in the individual disease entities and (ii) subgroups of patients who may be at increased risk of disease progression from exposure to air pollution and (iii) biomarkers capable of early identification of patients at higher risk for toxicity from pollution. By identifying air pollution as a potentially modifiable external risk factor for kidney disease progression that could be targeted on both a personal and community level (e.g., masks, air filters, behaviors, and public policies regarding clean air), the health and health outcomes could be improved for patients with primary glomerulopathies.

## Disclosure

Howard Trachtman reports employment with RenalStrategies LLC. He has active consultancy agreements with Aclipse, Alexion, Angion, Boerhinger-Ingelheim, Eloxx Pharmaceuticals, Goldfinch Bio, Maze Therapeutics, Natera (RenaSight), Otsuka (DSMB Chair for Pediatric Trials), PhaseV, ProKidney, Travere Therapeutics, Inc. and Walden; honoraria for participation in glomerular disease panels organized by Astellas and Reata; Steering Committee and Scientific Advisory Board for DUPRO (DUPLEX and POTECT Trials) for Travere Therapeutics, Inc.; member of the Kidney Health Initiative Board of Directors; Editorial board member of Pediatric Nephology, Glomerular Diseases, and Kidney360; serves as a partner with NephCure Kidney International in efforts to promote pediatric participation in clinical trials for glomerular diseases (PIONEER). Kretzler reports grants and contracts through the University of Michigan with the National Institutes of Health, Chan Zuckerberg Initiative, AstraZeneca, NovoNordisk, Eli Lilly, Gilead, Goldfinch Bio, Janssen, Boehringer-Ingelheim, Moderna, European Union Innovative Medicine Initiative, Certa, Chinook, amfAR, Angion, RenalytixAI, Travere, Regeneron, IONIS and Maze Therapeutics. He has received consulting fees through the University of Michigan from Astellas, Poxel, Janssen and UCB. Kretzler serves on the NIH-NCATS council and is on the board of Nephcure Kidney International. In addition, M.K. has a patent PCT/EP2014/073413 “Biomarkers and methods for progression prediction for chronic kidney disease” licensed. Mariani has grant funding from Boehringer-Ingelheim, Travere Therapeutics and Reliant Glycosciences. She consults Travere Therapeutics, Chinook Therapeutics and Calliditas Therapeutics. Ju has a patent PCT/EP2014/073413 “Biomarkers and methods for progression prediction for chronic kidney disease” licensed. Wenjun Ju reports grant from European Commission outside of the submitted work. William E. Smoyer is a cofounder of NephKey Therapeutics, Inc. and is on the Board of Directors of NephCure Kidney International and receives no compensation as a member of the Board of Directors. Kshirsagar receives royalties from UpToDate and has consulted Alkahest and Target RWE.

## Supplementary Material

Supplementary File (PDF) **Supplementary Acknowledgements:** a. CureGN; b. NEPTUNE. Figure S1. Spaghetti plots of individual patient exposure over time for PM_2.5_, (A), BC (B), and SO4 (C). **Table S1.** Summary statistics of demographic and clinical characteristics of NEPTUNE and CureGN participants. **Table S2.** Correlations between the exposures. **Table S3.** Descriptive characteristics in the overall cohort with Black Carbon (BC) exposure (μg/mm^3^) data available at baseline (*n* = 575) and divided into subgroups above and below the median level of exposure. **Table S4.** Descriptive characteristics in the overall cohort with sulfates (SO_4_) exposure (μg/mm^3^) data available at baseline (*n* = 575) and divided into subgroups above and below the median level of exposure. **Table S5.** Unadjusted and adjusted risk of ≥40% decline in eGFR or ESKD associated with untransformed levels of the 3 air pollution components in the pooled NEPTUNE and CureGN cohort. **Supplementary References.**

## Footnotes

Supplementary File (PDF)
**Supplementary acknowledgements:** a. CureGN; b. NEPTUNE **Supplementary Figure S1.** Spaghetti plots of individual patient exposure over time for PM2.5, (A), BC (B), and SO4 (C). **Supplementary Table S1.** Summary statistics of demographic and clinical characteristics of NEPTUNE and CureGN participants. **Supplementary Table S2.** Correlations between the exposures. **Supplementary Table S3.** Descriptive characteristics in the overall cohort with Black Carbon (BC) exposure (μg/mm^3^) data available at baseline (*n* = 575) and divided Into subgroups above and below the median level of exposure. **Supplementary Table S4.** Descriptive characteristics in the overall cohort with sulfates (SO_4_) exposure (μg/mm^3^) data available at baseline (*n* = 575) and divided into subgroups above and below the median level of exposure. **Supplementary Table S5.** Unadjusted and adjusted risk of ≥40% decline in eGFR or ESKD associated with untransformed levels of the 3 air pollution components in the pooled NEPTUNE and CureGN cohort.
**Supplementary References**
