# Supplementary material for: Elevated Exposure to Air Pollutants Accelerates Primary Glomerular Disease Progression
Source: Kidney Int Rep. 2024 May 18;9(8):2527–36. doi: 10.1016/j.ekir.2024.05.013 (PMC11328569; doi:10.1016/j.ekir.2024.05.013)

SUPPLEMENTAL MATERIALS

1. **SUPPLEMENTAL ACKNOWLEDGEMENTS**
    a. CureGN
    b. NEPTUNE
2. **SUPPLEMENTAL TABLES**

Supplemental Table S1. Summary statistics of demographic and clinical characteristics of NEPTUNE and CureGN participants

Supplemental Table S2. Correlations between the exposures

Supplemental Table S3. Descriptive characteristics in the overall cohort grouped by Black Carbon (BC) exposure at baseline

Supplemental Table S4. Descriptive characteristics in the overall cohort grouped by sulfates (SO4) exposure at baseline

Supplemental Table S5. Unadjusted and adjusted risk of ≥40% decline in eGFR or ESKD associated with untransformed levels of the three air pollution components in the pooled NEPTUNE and CureGN cohort

3. **SUPPLEMENTAL FIGURES**

Supplemental Figure 1. Spaghetti plots of individual patient exposure over time for PM2.5 (A), BC (B), and SO4 (C)

SUPPLEMENTAL ACKNOWLEDGEMENTS

Members of the Nephrotic Syndrome Study Network (NEPTUNE)
NEPTUNE Enrolling Centers
Atrium Health Levine Children's Hospital, Charlotte, SC: Susan Massengill*, Layla Lo[#]
Cleveland Clinic, Cleveland, OH: Katherine Dell*, John Sedor**, Stephanie Larson[#]
Children's Hospital, Los Angeles, CA: Ian Macumber* Kevin Lemley*, Silpa Sharma[#]
Children's Mercy Hospital, Kansas City, MO: Tarak Srivastava[*], Kelsey Markus[#]
Cohen Children's Hospital, New Hyde Park, NY: Christine Sethna[*], Suzanne Vento [#]
Columbia University, New York, NY: Pietro Canetta[*], Anup Pradhan[#]
Duke University Medical Center, Durham, NC: Opeyemi Olabasi**, Rasheed Gbadegesin*, Maurice Smith[#]
Emory University, Atlanta, GA: Laurence Greenbaum*, Chia-shi Wang*, Emily Yun[#]
The Lundquist Institute, Torrence, CA: Sharon Adler[*], Janine LaPage[#]
John H Stroger Cook County Hospital, Chicago, IL: Amatur Amarah*, Matthew Itteera[#]
Johns Hopkins Medicine, Baltimore, MD: Meredith Atkinson[*], Miahje Williams[#]
Mayo Clinic, Rochester, MN: John Lieske*, Marie Hogan**
Medical University of South Carolina, David Selewski[*], Cheryl Alston[#]
Montefiore Medical Center, Bronx, NY: Frederick Kaskel[*], Kim Reidy**, Michael Ross[*], Patricia Flynn[#]
NIDDK Intramural, Bethesda MD: Jeffrey Kopp[*]
New York University Medical Center, New York, NY: Laura Malaga-Dieguez[*], Olga Zhdanova**, Laura Jane Pehrson[#], Melanie Miranda[#]
The Ohio State University College of Medicine, Columbus, OH: Salem Almaani[*], Laci Roberts[#]
Stanford University, Stanford, CA: Richard Lafayette*, Shiktij Dave[#]
Temple University, Philadelphia, PA: Iris Lee[*], Zoey Pfeffer[#]
Texas Children's Hospital at Baylor College of Medicine, Houston, TX: Shweta Shah*, Aisha Deslandes[#] [#]
University Health Network Toronto: Heather Reich*, Michelle Hladunewich**, Paul Ling[#], Martin Romano[#]
University of California at San Francisco, San Francisco, CA: Paul Brakeman*
University of Colorado Anschutz Medical Campus, Aurora, CO: Amber Podoll* Nathan Rogers[#]
University of Kansas Medical Center, Kansas City, KS: Ellen McCarthy*, Elizabeth Landry[#]
University of Miami, Miami, FL: Alessia Fornoni[*], Carlos Bidot[#]
University of Michigan, Ann Arbor, MI: M Kretzler[*], Laura Mariani*, Zubin Modi*, A Williams[#], Meghan Stelzer[#]
University of Minnesota, Minneapolis, MN: Patrick Nachman*, Michelle Rheault*, Jenna Hanson[#]
University of North Carolina, Chapel Hill, NC: Vimal Derebail[*], Keisha Gibson[*], Anne Froment[#]
University of Pennsylvania, Philadelphia, PA: Lawrence Holzman[*], Kevin Meyers**, Krishna Kallem[#], Ann Swenson[#]
University of Texas San Antonio, San Antonio, TX: Samin Sharma[*]
University of Texas Southwestern, Dallas, TX: Elizabeth Roehm*, Kamalanathan Sambandam[*], Jamie Hellewege
University of Washington, Seattle, WA: Ashley Jefferson[*], Sangeeta Hingorani**, Katherine Tuttle**[§], Linda Manahan [#], Emily Pao[#], Kelli Kuykendall[§]
Wake Forest University Baptist Health, Winston-Salem, NC: Jen Jar Lin*
Washington University in St. Louis, St. Louis, MO: Ellen Cody*

Data Analysis and Coordinating Center: Matthias Kretzler*, Laura Barisoni**, Crystal Gadegbeku**, Brenda Gillespie**, Lawrence Holzman**, Laura Mariani**, Zubin Modi**,

Matthew G Sampson**, Eloise Salmon**, John Sedor**, Abigail Smith**, Howard Trachtman**, Jarcy Zee**, Gabrielle Alter, Hailey Desmond, Sean Eddy, Damian Fermin, Wenjun Ju, Maria Larkina, Shengqian Li, Shannon Li, Chrysta Lienczewski, Tina Mainieri, Rebecca Scherr, Jonathan Troost, Amanda Williams

Digital Pathology Committee: Carmen Avila-Casado (University Health Network, Toronto), Serena Bagnasco (Johns Hopkins University), Clarissa Cassol (Arakana), Lihong Bu (Mayo Clinic), Shelley Caltharp (Emory University), Dawit Demeke (University of Michigan), Brenda Gillespie (University of Michigan), Jared Hassler (Temple University), Leal Herlitz (Cleveland Clinic), Stephen Hewitt (National Cancer Institute), Jeff Hodgin (University of Michigan), Danni Holanda (Arkana), Neeraja Kambham (Stanford University), Kevin Lemley (Children's Hospital of Los Angeles), Laura Mariani (University of Michigan), Nidia Messias (Washington University), Alexei Mikhailov (Wake Forest), Behzad Najafian (University of Washington), Matthew Palmer (University of Pennsylvania), Avi Rosenberg (Johns Hopkins University), Virginie Royal (University of Montreal), Barry Stokes (Columbia University), David Thomas (Duke University), Michifumi Yamashita (Cedar Sinai), Hong Yin (Emory University) Jarcy Zee (University of Pennsylvania), Yiqin Zuo (University of Miami) Co-Chairs: Laura Barisoni (Duke University) and Cynthia Nast (Cedar Sinai).

CureGN Collaborators
The CureGN Consortium members listed below, from within the four Participating Clinical Center networks and Data Coordinating Center, are acknowledged by the authors as Collaborators.
**CureGN Principal Investigators; *CureGN Site Principal Investigators; #CureGN Lead Coordinators.

CureGN Participating Clinical Centers (PCC) through Columbia University:
Columbia University, New York, NY, US: Wooin Ahn, Gerald Appel, Paul Appelbaum, Revekka Babayev, Andrew Bomback, Pietro Canetta, Brenda Chan, Vivette Denise D'Agati, Samitri Dogra, Hilda Fernandez, Ali Gharavi**, William Hines, Syed Ali Husain, Namrata Jain, Krzysztof Kiryluk, Fangming Lin, Maddalena Marasa#, Glen Markowitz, Hila Milo Rasouly, Sumit Mohan, Nicola Mongera, Jordan Nestor, Thomas Nickolas, Jai Radhakrishnan, Maya Rao, Simone Sanna-Cherchi, Shayan Shirazian, Michael Barry Stokes, Natalie Uy, Anthony Valeri, Natalie Vena
University of Warsaw, Warszawa, Poland: Bartosz Foroncewicz, Barbara Moszczuk, Krzysztof Mucha*, Agnieszka Perkowska-Ptasińska
Gaslini Children's Hospital, Genoa, Italy: Gian Marco Ghiggeri*, Francesca Lugani
CureGN Participating Clinical Centers (PCC) through the Pediatric Nephrology Research Consortium:
Arkana Laboratories, Little Rock, AR, USA: Josephine Ambruzs, Helen Liapis
Children's Hospital of Michigan, Detroit, MI, USA: Rossana Baracco, Amrish Jain*
Children's Hospital of New Orleans/ LSU Health, New Orleans, LA, USA: Isa Ashoor, Diego Aviles*
Children's Mercy Hospital, Kansas City, MO, USA: Tarak Srivastava*
Children's National Medical Center, Washington DC, USA: Sun-Young Ahn*
Cincinnati Children's Hospital Cincinnati, OH, USA: Prasad Devarajan, Elif Erkan*, Donna Claes, Hillarey Stone
Connecticut Children's Medical Center, Hartford, CT, USA: Sherene Mason*
Duke Children's Hospital Medical Center, Durham, NC, USA: Rasheed Gbadegesin*
East Carolina University Brody School of Medicine, Greenville, NC, USA: Liliana Gomez-Mendez*
Emory University, Atlanta, GA, USA: Larry Greenbaum**, Chia-shi Wang, Hong (Julie) Yin
Helen DeVos Children's Hospital, Grand Rapids, MI, USA: Yi Cai*, Goebel Jens, Julia Steinke
Levine Children's Hospital/Atrium Health, Charlotte, NC, USA: Donald Weaver*
Lurie Children's Hospital, Chicago IL, USA: Jerome Lane*
Mayo Clinic, Rochester, MN, USA: Carl Cramer*
Medical College of Wisconsin, Milwaukee, WI, USA: Cindy Pan, Neil Paloian, Rajasree Sreedharan*
Medical University of South Carolina, Charleston SC, USA: David Selewski, Katherine Twombley*
Nationwide Children's Hospital, Columbus, OH, USA: Corinna Bowers#, Mary Dreher# Mahmoud Kallash*, John Mahan, Samantha Sharpe#, William Smoyer**
Oregon Health and Science University, Portland, OR, USA: Amira Al-Uzri*, Sandra Iragorri
Riley Children's Hospital, Indianapolis, IN, USA: Myda Khalid*
Cardinal Glennon Children's Medical Center/ St. Louis University, St. Louis, MO, USA: Craig Belsha*
Texas Children's Hospital, Houston, TX, USA: Joseph Alge*, Michael Braun, AC Gomez, Scott Wenderfer*
Texas Tech Health Sciences Center, Amarillo, TX, USA: Tetyana Vasylyeva*
Children's of Alabama, University of Alabama, Birmingham, AL, USA: Daniel Feig*
University of Colorado Children's Hospital, Colorado, Aurora, CO, USA: Gabriel Cara Fuentes, Melisha Hannah*
University of Iowa Children's Hospital, Iowa City, IA, USA: Carla Nester*
University of Kentucky, Lexington, KY, USA: Aftab Chishti*
University of Louisville, Louisville, KY, USA: Jon Klein**
Holtz Medical Center, University of Miami, Miami, FL, USA: Chryso Katsoufis, Wacharee Seeherunvong*
University of Minnesota Children's Hospital, Minneapolis, MN, USA: Michelle Rheault*

University of New Mexico Health Sciences Center, Albuquerque, NM, USA: Craig Wong*
University of Oklahoma Health Sciences Center, Oklahoma City, OK, USA: Nisha Mathews*
University of Virginia, Charlottesville, VA, USA: John Barcia*, Agnes Swiatecka-Urban
University of Wisconsin, Madison, WI, USA: Sharon Bartosh*
Vanderbilt Children's Hospital, Nashville TN, USA: Tracy Hunley*
Washington University in St. Louis, St. Louis, MO, USA: Vikas Dharnidharka*, Joseph, Gaut
CureGN Participating Clinical Centers (PCC) through the University of North Carolina:
Hôpital Maisonneuve-Rosemont, Montreal, Canada: Louis-Philippe Laurin*, Virginie Royal
Medical University of South Carolina, Charleston, SC, USA: Anand Achanti, Milos Budisavljevic*, Sally Self
Northwestern University, Chicago, IL, USA: Cybele Ghossein, Yonatan Peleg, Shikha Wadhwani*
Ohio State University, Columbus, OH, USA: Salem Almaani, Isabelle Ayoub, Tibor Nadasdy, Samir, Parikh, Brad Rovin*
University of Chicago, Chicago, IL, USA: Anthony Chang
University of Alabama at Birmingham, Birmingham, AL, USA: Huma Fatima, Bruce Julian, Jan Novak, Matthew Renfrow, Dana Rizk*
University of North Carolina Kidney Center, Chapel Hill, NC, USA: Dhruti Chen, Vimal Derebail, Ronald Falk**, Keisha Gibson, Dorey Glenn, Susan Hogan, Koyal Jain, J. Charles Jennette, Amy Mottl*, Caroline Poulton[#], Manish Kanti Saha
Vanderbilt University, Nashville, TN, USA: Agnes Fogo, Neil Sanghani*
Virginia Commonwealth University, Richmond, VA, USA: Jason Kidd*, Selvaraj Muthusamy
CureGN Participating Clinical Centers (PCC) through the University of Pennsylvania:
MetroHealth Medical Center/Case Western Reserve University, Cleveland, OH, USA: Jeffrey Schelling*
Cedars-Sinai Health System, Los Angeles, CA, USA: Jean Hou
Children's Hospital of LA, Los Angeles, CA, USA: Kevin Lemley*, Warren Mika, Pierre Russo
Children's Hospital of Philadelphia, Philadelphia, PA, USA: Michelle Denburg, Amy Kogon, Kevin Meyers*, Madhura Pradhan
Cleveland Clinic, Cleveland, OH, CA: Raed Bou Matar*, John O'Toole*, John Sedor*
Cohen Children's Medical Center, New Hyde Park, NY, USA: Christine Sethna*, Suzanne Vento [#]
Johns Hopkins University, Baltimore, MD, USA: Mohamed Atta, Serena Bagnasco, Alicia Neu, John Sperati*
Lundquist Institute at Harbor-UCLA Medical Center, Torrance, CA, USA: Sharon Adler*, Tiane Dai, Ram Dukkipati
Mayo Clinic, Rochester, MN, USA: Fernando Fervenza*, Sanjeev Sethi
Montefiore Medical Center, The Bronx, New York, NY, USA: Frederick Kaskel, Kaye Brathwaite, Kimberly Reidy*
New York University, New York, NY, USA: Joseph Weisstuch, Ming Wu, Olga Zhdanova
NIDDK, Bethesda, MD, USA: Jurgen Heymann, Jeffrey Kopp*, Meryl Waldman, Cheryl Winkler
Spokane Providence Medical Center, Spokane, WA, USA: Katherine Tuttle*
Stanford University, Palo Alto, CA, USA: Jill Krissberg, Richard Lafayette*, Kamal Fahmeedah, Elizabeth Talley
Sunnybrook Health Sciences Centre, Toronto, Canada: Michelle Hladunewich*
The Hospital for Sick Children, Toronto, Canada: Rulan Parekh*
University Health Network, Toronto, Canada: Carmen Avila-Casado, Daniel Cattran*, Reich Heather, Philip Boll
University of Miami, Miami, FL, USA: Yelena Drexler, Alessia Fornoni*
University of Michigan, Ann Arbor, MI, USA: Brooke Blazius*, Jeffrey Hodgin, Andrea Oliverio
University of Pennsylvania, Philadelphia, PA, USA: Jon Hogan, Lawrence Holzman**, Matthew Palmer, Gaia Coppock

University of Pittsburgh School of Medicine, Pittsburgh, PA, USA: Blaise Abromovitz*, Michael Mortiz*
University of Washington, Seattle, WA, USA: Charles Alpers, J. Ashley Jefferson*
UT Southwestern, Dallas, TX, USA: Elizabeth Brown, Kamal Sambandam*, Bethany Roehm

Data Coordinating Center (DCC):
Arbor Research Collaborative for Health, Ann Arbor, MI, USA: John Graff, Abigail Smith
Cedar Sinai Medical Center, Los Angeles, CA, USA: Cynthia Nast
Duke University, Durham, NC, USA: Laura Barisoni
University of Michigan, Ann Arbor, MI, USA: Brenda Gillespie**, Bruce Robinson**, Matthias Kretzler, Laura Mariani**

Steering Committee Chair: Lisa M. Guay-Woodford, Children's Hospital of Pennsylvania, Philadelphia, PA, USA

Supplemental Table S1. Summary statistics of demographic and clinical characteristics of NEPTUNE and CureGN participants

Characteristic	Overall (n=925)	NEPTUNE (n=228)	CureGN (n=697)	p-value
Age at baseline (years), median (IQR)	21.0 (9.0 to 48.0)	9.0 (3.5 to 31.0)	29.0 (13.0 to 50.0)	<0.001
Sex, n (%)				0.86
Male	516 (56)	126 (55)	390 (56)	
Female	409 (44)	102 (45)	307 (44)	
Race, n (%)				<0.001
Asian/Asian American	57 (6)	23 (10)	34 (5)	
Black/African American	204 (22)	53 (23)	151 (22)	
Multi-Racial	41 (4)	14 (6)	27 (4)	
Native Hawaiian/Other Pacific Islander	6 (1)	3 (1)	3 (0)	
White/Caucasian	584 (63)	121 (53)	463 (66)	
Unknown	33 (4)	14 (6)	19 (3)	
Ethnicity, n (%)				<0.001
Hispanic or Latino	157 (17)	46 (20)	111 (16)	
Not Hispanic or Latino	762 (82)	176 (77)	586 (84)	
Unknown	6 (1)	6 (3)	0 (0)	
Maternal education, n (%)				0.05
Unknown	92 (10)	32 (14)	60 (9)	
<High school	204 (22)	50 (22)	154 (22)	
High school	401 (43)	86 (38)	315 (45)	
>=4 year college	228 (25)	60 (26)	168 (24)	
Diagnosis, n (%)				<0.001
FSGS	203 (22)	28 (12)	175 (25)	
IgA nephropathy	202 (22)	2 (1)	200 (29)	
MCD	235 (25)	52 (23)	183 (26)	
MN	163 (18)	24 (11)	139 (20)	
Non-biopsied	106 (11)	106 (46)	0 (0)	
Other	16 (2)	16 (7)	0 (0)	
eGFR at baseline, median (IQR)	87.9 (57.3 to 111.7)	103.7 (79.6 to 120.0)	83.2 (52.5 to 105.5)	<0.001
UPCR at baseline, median (IQR)	1.8 (0.2 to 6.6)	6.8 (1.7 to 13.4)	1.2 (0.2 to 4.0)	<0.001
$PM_{2.5}$ at baseline, median (IQR)	8.0 (7.0 to 9.1)	8.3 (7.1 to 9.3)	7.9 (7.0 to 9.0)	0.01
Black carbon at baseline, median (IQR)	0.7 (0.6 to 0.9)	0.7 (0.6 to 0.9)	0.7 (0.6 to 0.8)	0.96
Sulfate (SO_4) at baseline, median (IQR)	1.5 (1.2 to 1.7)	1.4 (1.1 to 1.6)	1.5 (1.2 to 1.8)	0.01
Follow-up time (months), median (IQR)	49.0 (27.1 to 67.1)	30.0 (11.0 to 37.0)	58.9 (38.6 to 72.9)	<0.001

N missing per continuous variables: age=0, eGFR=53; UPCR=107; $PM_{2.5}$ =0; Black carbon=350; Sulfate=350; Follow-up time=0

Supplemental Table S2. Pearson correlation matrix of exposures.

	Particulate matter ($PM_{2.5}$)	Black carbon	Sulfates (SO_4)
$PM_{2.5}$	1.00	0.83	0.40
Black carbon	0.83	1.00	0.23
Sulfate (SO4)	0.40	0.23	1.00

All correlations were considered significant with $p<0.0001$.

Supplemental Table S3. Descriptive characteristics of participants in the overall cohort with available Black Carbon (BC) exposure data at baseline grouped below and above median exposure levels

Characteristic	Overall (n=575)	BC[a] ≤median (≤0.73) (n=287)	BC[a] >median (>0.73) (n=288)	p-value
$PM_{2.5}$ at baseline[a]	8.1 (7.1 to 9.2)	7.1 (6.3 to 7.7)	9.1 (8.4 to 10.2)	<0.001
Black carbon at baseline[a]	0.7 (0.6 to 0.9)	0.6 (0.5 to 0.7)	0.9 (0.8 to 1.0)	
Sulfate (SO_4) at baseline[a]	1.5 (1.2 to 1.7)	1.4 (1.0 to 1.6)	1.6 (1.3 to 1.9)	<0.001
Age at baseline (years)	23.0 (10.0 to 47.0)	16.0 (8.0 to 43.0)	29.0 (12.0 to 50.0)	<0.001
Sex, n (%)				0.22
Male	336 (58)	176 (61)	160 (56)	
Female	239 (42)	111 (39)	128 (44)	
Race, n (%)				<0.001
Asian/Asian American	36 (6)	14 (5)	22 (8)	
Black/African American	108 (19)	33 (11)	75 (26)	
Multi-Racial	25 (4)	10 (3)	15 (5)	
Native Hawaiian/Other Pacific Islander	3 (1)	2 (1)	1 (0)	
White/Caucasian	386 (67)	221 (77)	165 (57)	
Unknown	17 (3)	7 (2)	10 (3)	
Ethnicity, n (%)				0.002
Hispanic or Latino	94 (16)	38 (13)	56 (19)	
Not Hispanic or Latino	476 (83)	248 (86)	228 (79)	
Unknown	5 (1)	1 (0)	4 (1)	
Maternal education, n (%)				0.004
Unknown	46 (8)	16 (6)	30 (10)	
<High school	142 (25)	63 (22)	79 (27)	
High school	249 (43)	133 (46)	116 (40)	
≥4-year college	138 (24)	75 (26)	63 (22)	
Diagnosis, n (%)				0.17
FSGS	114 (20)	49 (17)	65 (23)	
IgA nephropathy	169 (29)	85 (30)	84 (29)	
MCD	139 (24)	75 (26)	64 (22)	
MN	90 (16)	45 (16)	45 (16)	
Non-biopsied	52 (9)	29 (10)	23 (8)	
Other	11 (2)	4 (1)	7 (2)	
eGFR at baseline[b]	86.2 (56.7 to 109.0)	89.2 (66.0 to 108.4)	81.1 (51.7 to 109.7)	<0.001
UPCR at baseline[c]	1.4 (0.2 to 5.0)	1.2 (0.2 to 5.0)	1.7 (0.3 to 4.8)	0.48
Follow-up time (months)	60.1 (29.0 to 75.2)	60.1 (30.0 to 73.9)	60.0 (27.0 to 77.8)	0.67

All values presented as median (IQR) unless otherwise specified.

Units [a]µg/mm^3; [b]ml/min/1.73 m2; [c]mg protein:mg creatinine

N missing per continuous variables: age=0, eGFR=30; UPCR=46; $PM_{2.5}$ =0; Black carbon=0; Sulfate=0; Follow-up time=0

Supplemental Table S4. Descriptive characteristics of participants in the overall cohort with available Sulfates (SO_4) exposure data at baseline grouped below and above median exposure levels

Characteristic	Overall (n=575)	SO_4^a ≤median (≤1.39) (n=287)	SO_4^a >median (>1.39) (n=288)	p-value
$PM_{2.5}$ at baseline[a]	8.1 (7.1 to 9.2)	7.4 (6.3 to 9.0)	8.5 (7.7 to 9.3)	<0.001
Black carbon at baseline[a]	0.7 (0.6 to 0.9)	0.7 (0.5 to 0.8)	0.8 (0.7 to 0.9)	<0.001
Sulfate (SO_4) at baseline[a]	1.5 (1.2 to 1.7)	1.2 (1.0 to 1.3)	1.7 (1.6 to 1.9)	
Age at baseline (years)	23.0 (10.0 to 47.0)	18.0 (9.0 to 43.0)	26.0 (11.0 to 50.5)	0.01
Sex, n (%)				0.45
Male	336 (58)	170 (59)	166 (58)	
Female	239 (42)	117 (41)	122 (42)	
Race, n (%)				0.004
Asian/Asian American	36 (6)	17 (6)	19 (7)	
Black/African American	108 (19)	39 (14)	69 (24)	
Multi-Racial	25 (4)	10 (3)	15 (5)	
Native Hawaiian/Other Pacific Islander	3 (1)	3 (1)	0 (0)	
White/Caucasian	386 (67)	205 (71)	181 (63)	
Unknown	17 (3)	13 (5)	4 (1)	
Ethnicity, n (%)				<0.001
Hispanic or Latino	94 (16)	69 (24)	25 (9)	
Not Hispanic or Latino	476 (83)	216 (75)	260 (90)	
Unknown	5 (1)	2 (1)	3 (1)	
Maternal education, n (%)				0.44
Unknown	46 (8)	31 (11)	15 (5)	
<High school	142 (25)	69 (24)	73 (25)	
High school	249 (43)	115 (40)	134 (47)	
≥4-year college	138 (24)	72 (25)	66 (23)	
Diagnosis, n (%)				0.07
FSGS	114 (20)	59 (21)	55 (19)	
IgA nephropathy	169 (29)	79 (28)	90 (31)	
MCD	139 (24)	73 (25)	66 (23)	
MN	90 (16)	37 (13)	53 (18)	
Non-biopsied	52 (9)	32 (11)	20 (7)	
Other	11 (2)	7 (2)	4 (1)	
eGFR at baseline[b]	86.2 (56.7 to 109.0)	89.2 (60.3 to 112.4)	83.4 (54.3 to 103.5)	0.001
UPCR at baseline[c]	1.4 (0.2 to 5.0)	1.6 (0.2 to 5.0)	1.3 (0.2 to 4.4)	0.32
Follow-up time (months)	60.1 (29.0 to 75.2)	51.8 (29.0 to 71.5)	66.2 (27.8 to 78.9)	0.05

All values presented as median (IQR) unless otherwise specified.

Units [a]μg/mm^3; [b]ml/min/1.73 m2; [c]mg protein:mg creatinine

N missing per continuous variables: age=0, eGFR=30; UPCR=46; $PM_{2.5}$ =0; Black carbon=0; Sulfate=0; Follow-up time=0

Supplemental Table S5. Unadjusted and adjusted risk of ≥40% decline in eGFR or ESKD associated with untransformed levels of the three air pollution components in the pooled NEPTUNE and CureGN cohort

**POOLED (NEPTUNE+CureGN)**	**$PM_{2.5}$ ($\mu g/mm^3$) Estimate [95%CI]**	**p-value**	**Black carbon ($\mu g/mm^3$) Estimate [95%CI]**	**p-value**	**Sulfates ($\mu g/mm^3$) Estimate [95%CI]**	**p-value**
Unadjusted						
**Environmental marker at baseline (per 1 SD)**	**1.55 [1.00, 2.38]**	**0.0489**	**1.43 [0.98, 2.07]**	**0.0026**	**1.08 [0.91, 1.27]**	**0.3706**
Adjusted-1						
**Environmental marker at baseline (per 1 SD)**	**1.16 [1.01, 1.34]**	**0.0424**	**1.23 [1.03, 1.48]**	**0.0233**	**1.03 [0.86, 1.24]**	**0.7269**
Age (per 1 year)	1.00 [0.99, 1.01]	0.7307	1.01 [1.00, 1.02]	0.0590	1.01 [1.00, 1.02]	0.0440
Race: Black vs. non-black	1.61 [1.17, 2.22]	0.0033	2.20 [1.47, 3.30]	0.0001	2.28 [1.52, 3.43]	<.0001
Maternal education						
High school vs. <High school	1.52 [1.07, 2.15]	0.0199	1.98 [1.26, 3.13]	0.0031	1.74 [1.13, 2.69]	0.0119
College vs. <High school	0.83 [0.53, 1.29]	0.3985	1.15 [0.64, 2.04]	0.6446	1.01 [0.58, 1.78]	0.9647
Adjusted-2						
**Environmental marker at baseline (per 1 SD)**	**1.15 [0.99, 1.32]**	**0.0602**	**1.19 [1.00, 1.42]**	**0.0445**	**1.00 [0.83, 1.21]**	**0.9767**
Age (per 1 year)	0.99 [0.98, 1.00]	0.0015	0.99 [0.98, 1.00]	0.1697	0.99 [0.98, 1.00]	0.1872
eGFR at baseline (per 1 ml)*	0.98 [0.98, 0.99]	<.0001	0.98 [0.97, 0.99]	<.0001	0.98 [0.97, 0.99]	<.0001
Race: Black vs. non-black	1.28 [0.92, 1.78]	0.1381	1.71 [1.12, 2.60]	0.0132	1.72 [1.13, 2.63]	0.0120
Maternal education						
High school vs. <High school	1.46 [1.03, 2.08]	0.0332	1.83 [1.16, 2.88]	0.0088	1.64 [1.06, 2.52]	0.0257
College vs. <High school	0.84 [0.54, 1.31]	0.4309	1.12 [0.63, 1.98]	0.7058	1.01 [0.58, 1.78]	0.9600

Supplementary Figure S1. Spaghetti plots of individual patient exposure over time for a. particulate matter (PM2.5); b. black carbon; and c. sulfates

a.

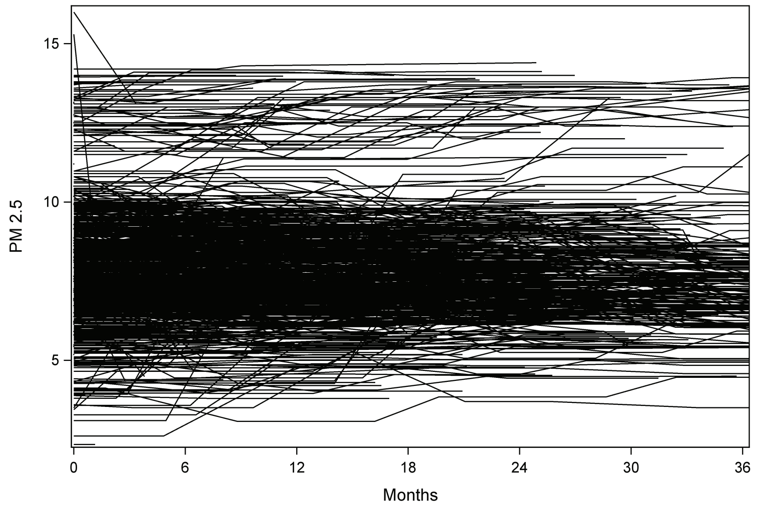

b.

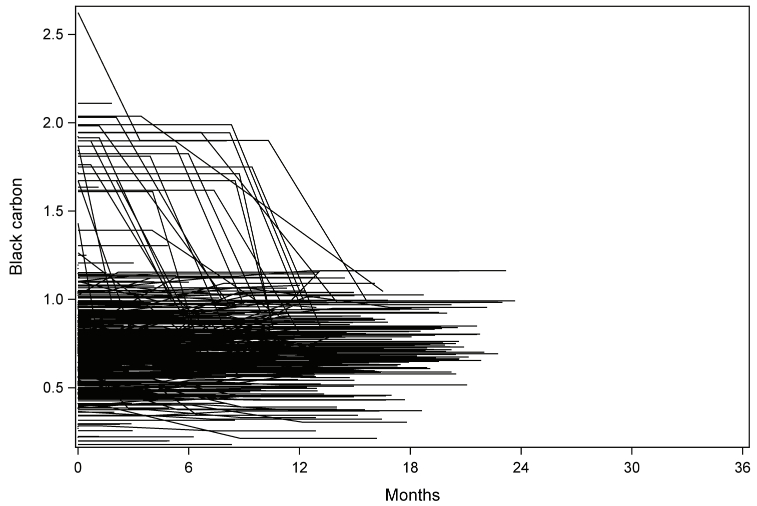

c.

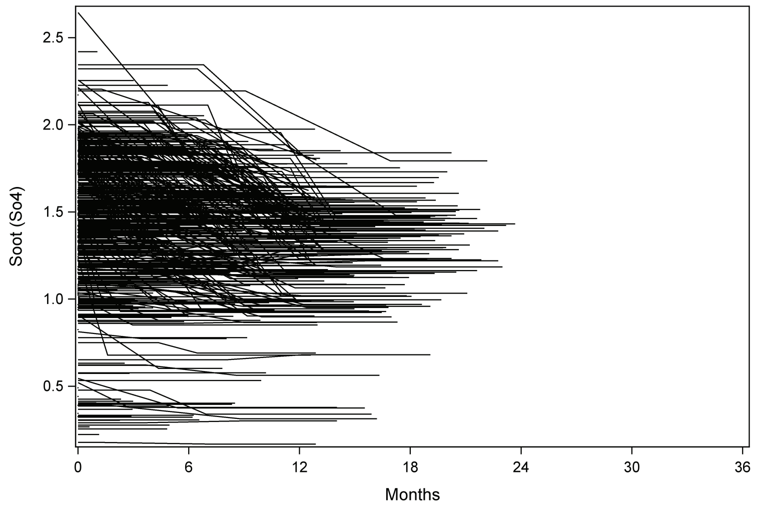

Supplement: Supplementary File (PDF) — Supplementary Acknowledgements: a. CureGN; b. NEPTUNE. Figure S1. Spaghetti plots of individual patient exposure over time for PM2.5, (A), BC (B), and SO4 (C). Table S1. Summary statistics of demographic and clinical characteristics of NEPTUNE and CureGN participants. Table S2. Correlations between the exposures. Table S3. Descriptive characteristics in the overall cohort with Black Carbon (BC) exposure (μg/mm3) data available at baseline (n = 575) and divided into subgroups above and below the median level of exposure. Table S4. Descriptive characteristics in the overall cohort with sulfates (SO4) exposure (μg/mm3) data available at baseline (n = 575) and divided into subgroups above and below the median level of exposure. Table S5. Unadjusted and adjusted risk of ≥40% decline in eGFR or ESKD associated with untransformed levels of the 3 air pollution components in the pooled NEPTUNE and CureGN cohort. Supplementary References. [file mmc1.pdf]
